# Identification of mutations in the PI3K-AKT-mTOR signalling pathway in patients with macrocephaly and developmental delay and/or autism

**DOI:** 10.1186/s13229-017-0182-4

**Published:** 2017-12-20

**Authors:** Kit San Yeung, Winnie Wan Yee Tso, Janice Jing Kun Ip, Christopher Chun Yu Mak, Gordon Ka Chun Leung, Mandy Ho Yin Tsang, Dingge Ying, Steven Lim Cho Pei, So Lun Lee, Wanling Yang, Brian Hon-Yin Chung

**Affiliations:** 1 Department of Paediatrics and Adolescent Medicine, The University of Hong Kong, Pok Fu Lam, Hong Kong, China; 2 Department of Paediatrics and Adolescent Medicine, The Duchess of Kent Children’s Hospital, Pok Fu Lam, Hong Kong, China; 3 Department of Radiology, Queen Mary Hospital, Room 103, New Clinical Building, 102 Pokfulam Road, Pok Fu Lam, Hong Kong, China

**Keywords:** Somatic mosaicism, Macrocephaly, Megalencephaly, Developmental delay, Autism spectrum disorder, *PTEN*, *MTOR*, *PIK3CA*, *PPP2R5D*

## Abstract

**Background:**

Macrocephaly, which is defined as a head circumference greater than or equal to + 2 standard deviations, is a feature commonly observed in children with developmental delay and/or autism spectrum disorder. Although *PTEN* is a well-known gene identified in patients with this syndromic presentation, other genes in the PI3K-AKT-mTOR signalling pathway have also recently been suggested to have important roles. The aim of this study is to characterise the mutation spectrum of this group of patients.

**Methods:**

We performed whole-exome sequencing of 21 patients with macrocephaly and developmental delay/autism spectrum disorder. Sources of genomic DNA included blood, buccal mucosa and saliva. Germline mutations were validated by Sanger sequencing, whereas somatic mutations were validated by droplet digital PCR.

**Results:**

We identified ten pathogenic/likely pathogenic mutations in *PTEN* (*n* = 4), *PIK3CA* (*n* = 3), *MTOR* (*n* = 1) and *PPP2R5D* (*n* = 2) in ten patients. An additional *PTEN* mutation, which was classified as variant of unknown significance, was identified in a patient with a pathogenic *PTEN* mutation, making him harbour bi-allelic germline *PTEN* mutations. Two patients harboured somatic *PIK3CA* mutations, and the level of somatic mosaicism in blood DNA was low. Patients who tested positive for mutations in the PI3K-AKT-mTOR pathway had a lower developmental quotient than the rest of the cohort (DQ = 62.8 vs. 76.1, p = 0.021). Their dysmorphic features were non-specific, except for macrocephaly. Among the ten patients with identified mutations, brain magnetic resonance imaging was performed in nine, all of whom showed megalencephaly.

**Conclusion:**

We identified mutations in the PI3K-AKT-mTOR signalling pathway in nearly half of our patients with macrocephaly and developmental delay/autism spectrum disorder. These patients have subtle dysmorphic features and mild developmental issues. Clinically, patients with germline mutations are difficult to distinguish from patients with somatic mutations, and therefore, sequencing of buccal or saliva DNA is important to identify somatic mosaicism. Given the high diagnostic yield and the management implications, we suggest implementing comprehensive genetic testing in the PI3K-AKT-mTOR pathway in the clinical evaluation of patients with macrocephaly and developmental delay and/or autism spectrum disorder.

**Electronic supplementary material:**

The online version of this article (10.1186/s13229-017-0182-4) contains supplementary material, which is available to authorized users.

## Background

Macrocephaly is defined as a disproportionally enlarged head size with an occipitofrontal circumference greater than or equal to +2 standard deviations (SDs). On the other hand, megalencephaly is defined as hyperplasia of the brain parenchyma observed in a radiological examination together with clinical features of macrocephaly. Both conditions are associated with developmental delay (DD) and/or autism spectrum disorder (ASD). Moreover, a recent neuroimaging study has shown that brain volume overgrowth is linked to the emergence and severity of autistic social deficits [1].

ASD is a complex, behaviourally defined disorder characterised by impairments in communication and reciprocal social interaction, restrictive interests and repetitive stereotypic behaviours [2]. It is known that ASD has a strong genetic basis [3, 4], and environmental factors might also influence the development of ASD [5–7]. Previous studies have reported a genetic diagnosis in 10% to 40% of patients with ASD [8–11]. According to the American Academy of Pediatrics guidelines for ASD published in 2000, genetic testing is a standard diagnostic test for children with ASD and dysmorphic features or intellectual disability (ID).

As shown in previous studies, 14–34% of children with ASD also have macrocephaly [12–17], and a meta-analysis revealed that 15.7% have macrocephaly and 9.1% have brain overgrowth [18]. *PTEN* is a well-known gene associated with ASD and macrocephaly [19–21]. Hence, genetic testing for *PTEN* mutations is recommended as part of the clinical evaluation in this group of patients [22–24]. Recently, mutations in other genes in the PI3K-AKT-mTOR signalling pathway, including *PIK3CA*, *PIK3R2*, *MTOR*, *CCND2* and *PPP2R5D*, were also reported in patients with ASD/DD and macrocephaly [25–30]. Although most *PTEN* mutations reported in this group of patients were germline mutations [20, 31], mutations in other genes in the PI3K-AKT-mTOR signalling pathway were frequently detected with a low level of mosaicism, which are undetectable using conventional Sanger sequencing. The use of next-generation sequencing, such as whole-exome sequencing (WES) or target panel sequencing, enables the detection of the low level of mosaicism in these patients. In this study, we aim to define the mutation spectrum in a cohort of patients with ASD/DD and macrocephaly using WES.

## Methods

### Patient recruitment

We recruited patients from January 2013 to December 2016 at the Duchess of Kent Children’s Hospital Child Assessment Center (DKCAC). Patients were initially assessed by a developmental paediatrician and relevant allied health professionals, including clinical psychologists, physiotherapists, occupational therapists and speech therapists. The developmental profile of patients less than 72 months of age was assessed using the Griffiths Mental Developmental Scales-Extended Revised (GMDS-ER). The developmental quotient (DQ) score was calculated based on the neurodevelopmental assessment to compare the developmental profiles between mutation-positive and mutation-negative patients. Because many patients were examined using more than one developmental assessment, the DQ scores from the first assessment were used. DQ scores were estimated for four patients based on their allied health assessment records and a clinical assessment by a developmental paediatrician because the patient was either too sick for the formal assessment with GMDS-ER (patient 3) or data were missing (patients 5, 15 and 19). Intellectual functioning of children ≥72 months of age was assessed using the Hong Kong Wechsler Intelligence Scale. ASD was diagnosed based on the Diagnostic and Statistical Manual of Mental Disorders—Fourth Edition (DSM-IV) criteria for ASD. Suspected cases were further assessed using the Autism Diagnostic Observation schedule (ADOS).

Developmental paediatricians at DKCAC referred patients with negative findings in the chromosomal microarray to the clinical geneticist when the patients also presented with macrocephaly (head circumference ≥ + 2 SD). These patients were recruited by the clinical geneticist, with the exception of patients presenting with obvious syndromic diseases who were assessed using targeted genetic tests and excluded from this study. Twenty-one unrelated patients were recruited in this study. Anthropometric data used for the measurements are specific to Hong Kong. Buccal swab or saliva sample was obtained in addition to the blood sample from five patients. This study was approved by the Institutional Review Board of the University of Hong Kong/Hospital Authority Hong Kong West Cluster (UW 12–211), and written consent was obtained from the patients’ parents.

### WES

WES was first performed at Macrogen Inc. and then at our university at a later stage of the project using either a TruSeq Exome Enrichment Kit (*n* = 9), SeqCap EZ Exome + UTR Exome Kit (*n* = 9) or TruSeq Rapid Exome Library Prep Kit (*n* = 3). The choice of the enrichment kit depended on the library preparation kit available at the time of sequencing. We aimed to obtain a minimum of 50X depth for WES performed with genomic DNA obtained from the blood and a minimum of 100X depth for WES performed with DNA derived from buccal mucosa or saliva samples. The increased sequencing depth in buccal and saliva samples served to increase the chance of detecting somatic mutations. Details of the library preparation method, sequencer used and average depth after sequence alignment of each individual is presented in Additional file 1.

### Variant calling and data analysis

Raw reads were aligned to the UCSC hg19 reference human genome using BWA 0.5.7, and variant calling was performed according to the best practices of GATK 3.4. The output VCF files were than annotated using ANNOVAR. The following criteria were applied to filter the variants and generate a list of high-quality variants for subsequent analyses: variants located outside the exonic regions and splice sites, synonymous variants, population frequency ≥ 1% or total depth < 10X. Genes involved in the PI3K-AKT-mTOR signalling pathway (*PTEN*, *AKT1*, *AKT3*, *PIK3CA*, *MTOR*, *PIK3R2*, *CCND2*, *PPP2R5D* and *PPP2R1A*) were prioritised for analysis because of their known pathogenicity in patients with macrocephaly and DD/ASD, and other genes in the PI3K-AKT signalling pathway obtained from Kyoto Encylopedia of Genes and Genomes (KEGG; reference: hsa04151) were also analysed. Mutations identified in this pathway that are associated with diseases are expected to be rare, and activating mutations are likely to be found in the Catalogue of Somatic Mutations in Cancer (COSMIC) database [27, 32]. In addition, genes involved in epigenetic regulation have recently been reported to be an important cause of macrocephaly/overgrowth and DD/ASD, and thus, genes related to epigenetic regulation (*CHD8*, *DNMT3A*, *EED*, *EZH2*, *HIST1H1E* and *NSD1*) were also prioritised for analysis [33].

### Confirmation of variants

For germline mutations, variants were confirmed by Sanger sequencing, and parental sequencing was also performed to determine whether the mutation was de novo or inherited. For somatic mosaic mutations, droplet digital PCR was used for validation, as described previously [32].

### Statistical analysis

Unpaired *t* tests were performed to assess the significance between the DQ scores of patients with and without mutations. A *p*-value < 0.05 was considered statistically significant. Analyses were performed using SPSS Statistics version 19 (IBM).

## Results

**Fig. 1:**
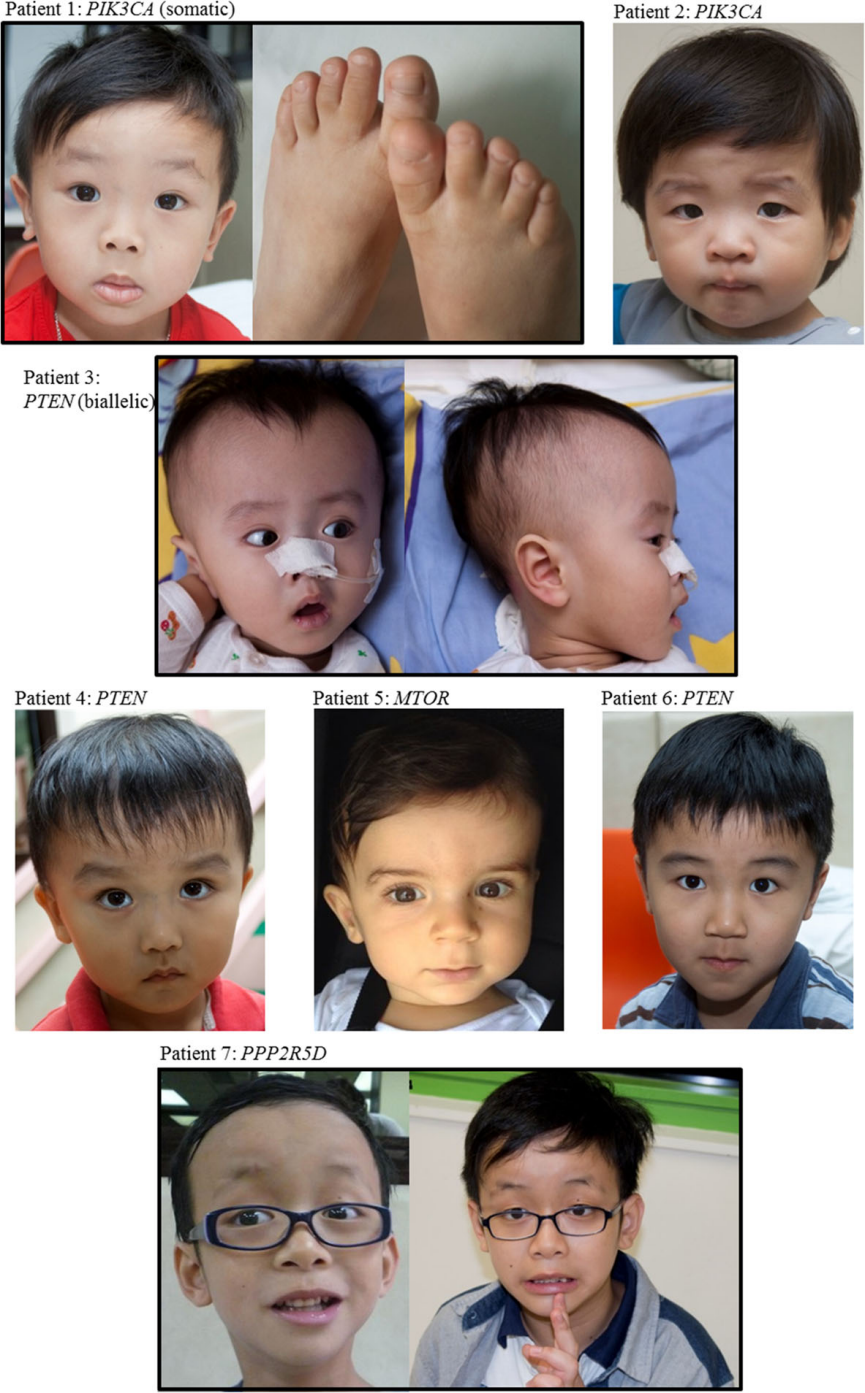
Clinical photographs of patients with mutations in the PI3K-AKT-mTOR pathway. Clinical photographs of patients with mutations identified in the PI3K-AKT-mTOR pathway are shown. For patient 1, syndactyly is shown. Patient 7 presented with hypertelorism and frontal bossing

Twenty-one patients (17 males and 4 females, 4 to 108 months of age at the time of clinical assessment/recruitment) with macrocephaly and DD/ID/ASD were recruited. All patients had DD at the time of recruitment, and three patients were diagnosed with ID in subsequent assessments. Among the 21 patients, ten were also diagnosed with ASD and two with suspected ASD (i.e., patients with autistic features who had not yet satisfied all the DSM-IV criteria for a diagnosis of ASD). A summary of the patients’ clinical presentations is presented in Table 1. Prior to WES, the chromosomal microarray was performed on these patients, as described previously [34], and no pathogenic/likely pathogenic copy number variations were identified in these patients. WES identified ten pathogenic/likely pathogenic mutations in ten patients (Fig. 1, Table 2), corresponding to a diagnostic yield of 47.6%. All pathogenic mutations were located in genes involved in the PI3K-AKT-mTOR signalling pathway, including *PTEN* (*n* = 4), *PIK3CA* (*n* = 3), *MTOR* (*n* = 1) and *PPP2R5D* (*n* = 2). Although most variants were germline mutations, two somatic *PIK3CA* mutations were identified. No pathogenic mutations were identified in genes related to epigenetic regulation, such as *CHD8*, *DNMT3A*, *EED* and *NSD1*, as reported by Tatton-Brown K et al. [33], and the analysis of the remainder of the exome did not reveal other variants of interest.

**Table 1:** Clinical presentations of all patients recruited in this study

Patient ID	Sex	First assessment (years)	Most recent assessment (years)	HC (z-score)	DQ	ASD	Developmental problem(s)	Other clinical features	MRI findings	Gene
1	M	3.0	5.2	3.6	75		Mild GDD with severe language delay	2/3/4 syndactyly	MEG, PG, VM, PWMSA	*PIK3CA*
2	M	1.5	4.1	3.2	55		Moderate GDD	Mild 2/3 syndactyly	MEG, PG, PWMSA	*PIK3CA*
3	M	0.8	1.6	5.9	50*		Moderate GDD	Recurrent respiratory infections, hypoglycaemia	MEG, PG, VM, PWMSA	*PTEN*
4	M	2.3	6.5	4.0	73	Suspected ASD	Mild GDD	NA	MEG, PG, PWMSA	*PTEN*
5	M	0.4	0.7	6.7	62*		Mild GDD	Liver haemangioma	MEG, PG, VM, PWMSA	*MTOR*
6	M	2.7	8.2	4.5	80	ASD	Mild GDD	2/3 syndactyly	NA	*PTEN*
7	M	2.0	11.6	2.6	49	ASD	Moderate GDD, mild ID	Hypertelorism, epilepsy^a^	MEG	*PPP2R5D*
8	M	1.8	5.3	2.2	54	Suspected ASD	Moderate GDD, mild ID	Hypertelorism, epilepsy^b^	MEG	*PPP2R5D*
9	M	2.0	3.5	3.5	62	ASD	Mild GDD with moderate language delay	Left lower limb hypertrophy with 2/3 syndactyly	MEG	*PIK3CA*
10	M	2.6	6.0	3.4	68		Mild GDD	Frontal bossing	MEG	*PTEN*
11	M	4.4	10.1	3.4	95	ASD	Language delay	z-scores for height: 3.0; weight: 5.6	Normal	Negative
12	M	2.3	6.3	3.3	79	ASD	Borderline delay with language delay	Left ptosis, scoliosis	Dilated cisterna magna, relative atrophy of the right cerebellar hemisphere	Negative
13	F	9.8	10.3	3.1	NA	ASD		NA	Normal	Negative
14	F	1.6	7.3	3.3	75		Borderline delay with language delay	Intra-cranial haemorrhage in the perinatal period	Aqueduct stenosis with hydrocephalus	Negative
15	F	4.0	6.0	4.2	90*	ASD	Language delay	NA	NA	Negative
16	M	2.2	23.4	3.2	71	ASD	Borderline delay	NA	NA	Negative
17	M	1.8	14.4	4.8	84	ASD	Borderline delay	Epilepsy^c^	Normal	Negative
18	F	2.4	6.2	2.2	78		Borderline delay	NA	NA	Negative
19	M	2.0	19.8	2.1	50*	ASD	Moderate GDD, moderate ID	Epilepsy^d^	NA	Negative
20	M	2.0	2.3	2.8	67		Mild GDD	NA	Dilated ventricles with external hydrocephalus	Negative
21	M	2.4	10.8	3.0	72		Mild GDD	Cleft lip and palate, nasal deformity	Normal	Negative

^*^Estimated DQ scores

^a^Epilepsy: onset at 4 years of age, generalised tonic-clonic seizure

^b^Epilepsy: onset at 4 days of age, generalised epileptic spasm

^c^Epilepsy: onset at 11 months, focal seizure with impaired awareness

^d^Epilepsy: onset 11 years of age, generalised tonic-clonic seizure

*MEG* megalencephaly, *PG* polymicrogyria, *VM* ventriculomegaly, *PWMSA* periventricular white matter signal abnormalities, *NA* not available

**Table 2:** Mutations in genes involved in the PI3K-AKT-mTOR pathway identified in the ten patients with macrocephaly and DD/ASD

Patient ID	Gene	Nucleotide change	Amino acid change	Inheritance	Mutation type	Allelic count in ExAC	No. of cases in COSMIC	Reported mutation
1	*PIK3CA*	c.G263A	p.(Arg88Gln)	De novo	Mosaic	0	137	43
2	*PIK3CA*	c.G1030A	p.(Val344Met)	Maternal	Germline	0	15	27
3	*PTEN*	c.G314T	p.(Cys105Phe)	De novo	Germline	0	6	39b
		c.G492T	p.(Lys164Asn)	Maternal	Germline	1 in 120,466	0^b^	
4	*PTEN*	c.G509C	p.(Ser170Thr)	De novo	Germline	0	0^b^	35.36^b^
5^a^	*MTOR*	c.G5395A	p.(Glu1799Lys)	De novo	Germline	0	10	28.45
6	*PTEN*	c.546delA	p.(Lys183ArgfsTer16)	De novo	Germline	0	0^b^	
7	*PPP2R5D*	c.G592A	p.(Glu198Lys)	De novo	Germline	0	1	29.46
8	*PPP2R5D*	c.G592A	p.(Glu198Lys)	De novo	Germline	0	1	29.46
9	*PIK3CA*	c.G2740A	p.(Gly914Arg)	De novo	Mosaic	0	2	25.27
10	*PTEN*	c.A203G	p.(Tyr68Cys)	De novo	Germline	0	4	37.38

The COSMIC database (June 2017) was accessed to retrieve the number of somatic mutations identified in cancer samples

^a^This patient was reported in a previous study [28] with the patient ID LR15-065

^b^Other mutations in the same codon have been reported

Mutations in the *PTEN* gene were the most frequently identified mutations in our patients, with four pathogenic variants found in four patients (19% among 21 patients). Both missense and frameshift mutations were identified. Although the *PTEN* p.(Ser170Thr) mutation detected in patient 4 has not previously been reported, a mutation in the same codon, resulting in *PTEN* p.(Ser170Arg), has been reported in multiple patients with *PTEN* cancer syndrome [35, 36], suggesting the pathogenicity of mutations in this amino acid. The mutation in patient 6 was a frameshift mutation and therefore a pathogenic mutation, because loss of function mutations is known to cause disease. The *PTEN* p.(Tyr68Cys) mutation identified in patient 10 has already been reported in multiple patients with Cowden Syndrome [37, 38].

In addition, we report here a second patient with bi-allelic germline *PTEN* mutations. Two *PTEN* mutations were identified in patient 3, where p.(Cys105Phe) was a de novo mutation and p.(Lys164Asn) was maternally inherited. Based on the sequencing data, the two mutations did not occur in the same allele (Additional file 2, Fig. S1a). Exon 5 was cloned to confirm that the mutations were located on different alleles, and clonal sequencing showed that the two mutations occurred on different alleles (Additional file 2, Fig. S1b). The mutation p.(Cys105Phe) has not been reported, but a mutation in the same codon resulting in p.(Cys105Tyr) has been reported in patients with Bannayan-Riley-Ruvalcaba Syndrome [39], suggesting the pathogenicity of mutations in this amino acid. The maternally inherited p.(Lys164Asn) mutation has not been reported in a disease-specific database and has only been reported in the Exome Aggregation Consortium (ExAC) database with an allelic frequency of 1 in 120,466. Familial testing showed that this mutation was also detected in the patient’s mother and elder sister, and both the mother and elder sister had macrocephaly (z-scores for the head circumference were 2.6 and 3.7, respectively). Both had unremarkable developmental issues. Active cancer surveillance was recommended, and at the age of 38, the patient’s mother was diagnosed with multifocal papillary carcinoma. Based on the above evidence, although the variant p.(Lys164Asn) being pathogenic was compelling, it was still classified as a variant of unknown significance. It was because the mother did not meet the diagnostic criteria for *PTEN* hamartoma tumour syndrome, who only fulfilled one major criterion (macrocephaly) and one minor criterion (papillary carcinoma) [40]. Patient 3 who had bi-allelic mutations, however, displayed a severe clinical presentation despite one of the mutation being classified as a variant of unknown significance. In addition to megalencephaly, polymicrogyria and developmental delay, he suffered from recurrent sinopulmonary infections and colitis, resulting in persistent fever and septic shock that required care in the intensive care unit. The immune workup showed hypogammaglobulinaemia, specifically, a low level of IgG subclass 3. During a salmonella gastrointestinal infection at 19 months of age, a dihydrorhodamine test showed a suppressed oxidative burst with only half of the function compared to the control. However, a specific primary immunodeficiency syndrome was not identified. Second, this patient had suffered from recurrent hypoglycaemia since 19 months of age that required high glucose infusions; however, his insulin level was normal and an extensive endocrine workup was unremarkable. The patient died at 25 months of age due to sepsis. This case showed that patients with bi-allelic *PTEN* mutations can present with other PI3K-AKT-mTOR pathway-related features, including the recurrent respiratory infections observed in patients with *PIK3CD* mutations [41, 42] and hypoglycaemia observed in patients with *AKT2* or *AKT3* mutations [43, 44].

The *PIK3CA* mutation was the second most common mutation identified in our patients (patients 1, 2 and 9). The germline mutation in patient 2 was inherited from his mother, who had macrocephaly (z-score of head circumference was 4.6) but no history of developmental issues. In addition to the germline mutation, two somatic mutations were identified, and all mutations have been reported previously [25, 27]. WES detected a p.(Arg88Gln) mutation in patient 1, with a percentage of 4.5% (4 of 89 reads) in the blood and 27.1% (29 of 107 reads) in the buccal mucosa, whereas confirmation using droplet digital PCR showed that the percentages of p.(Arg88Gln) mutations in the blood and buccal mucosa samples were 8.6 and 22.8%, respectively. For patient 9, WES detected a p.(Gly914Arg) mutation with a percentage of 2.8% (3 of 109 reads) in the blood and 11.9% (13 of 109 reads) in the saliva. Again, droplet digital PCR confirmed the WES results, showing that the percentages of mutations were 2.6, 9.3 and 22.8% in the blood, saliva and buccal mucosa samples from patient 9, respectively. Our results confirmed previous findings that the mutation load in saliva or buccal mucosa is higher than the mutation load in the blood [25–27].

Finally, known pathogenic variants in the *MTOR* [28, 45] and *PPP2R5D* [29, 46] genes were also identified in our patients. Patient 5, who had a *MTOR* mutation, has already been reported in another publication (referred to as LR15-065 in the publication) describing a wide spectrum of patients with germline/somatic *MTOR* mutations [28]. In addition, *PPP2R5D* p.(Glu198Lys) was identified twice in two unrelated patients as de novo mutation. Both patients had a clinical presentation compatible with other patients with *PPP2R5D* mutations, including hypertelorism, frontal bossing, and a history of epilepsy.

**Fig. 2:**
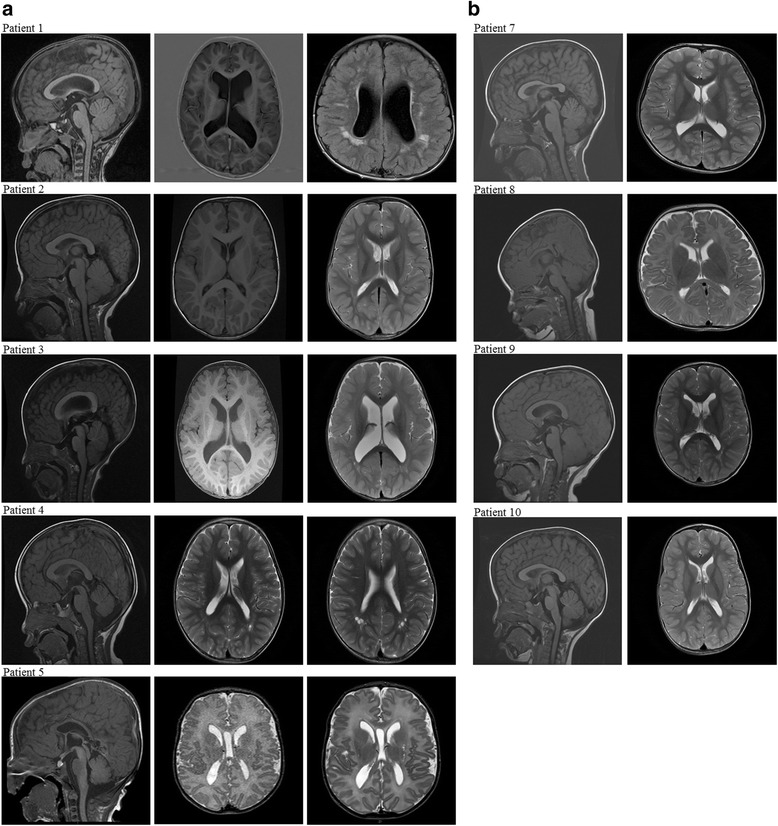
MRI of patients with mutations in the PI3K-AKT-mTOR pathway. **a** MRI in patients 1–5 showing megalencephaly, polymicrogyria and periventricular white matter signal abnormalities. Ventriculomegaly was observed in patients 1, 3 and 5. **b** MRI in patients 7–10 showing megalencephaly, without other abnormalities. MRI was not available for patient 6 because the family declined the MRI

At the time when genetic counselling was provided to patients with mutations, patients were re-examined to determine whether they had features of *PTEN* hamartoma tumour syndrome [40] such as macular pigmentation of the glans penis, mucocutaneous lesions and lipomas. For megalencephaly-capillary malformation syndrome (MCAP)/megalencephaly-polymicrogyria-polydactyly-hydrocephalus syndrome (MPPH) [47], features of syndactyly, signs of overgrowth and vascular anomalies were assessed. Most of these features were absent in our patients. Only a small minority of patients presented with additional clinical features, such as syndactyly, hypertelorism and epilepsy. Our findings demonstrated the diversity of the clinical spectrum in this group of patients (see Table 1). Overall, patients with identified mutations had only subtle dysmorphic features (Fig 1). The mean DQ scores for mutation-positive and mutation-negative patients were 62.8 and 76.1, respectively, and the difference was statistically significant (*p* = 0.021). The prevalence of ASD/autistic features was similar between the two groups. It was noted that except for the two patients with *PPP2R5D* mutations, the head circumference of other mutation-positive patients were > + 3 SD. However, patients who tested positive for mutations in the PI3K-AKT-mTOR pathway did not had a significant difference in head circumference than mutation-negative patients. We also reviewed the brain magnetic resonance imaging (MRI) findings. Of the ten patients with an identified mutation, nine underwent MRI (Fig. 2). Megalencephaly was present in all nine of these patients; in addition, polymicrogyria was also identified in five patients, periventricular white matter signal abnormalities were identified in five patients, and ventriculomegaly was identified in three patients. With the exception of brain overgrowth, mutation-positive patients had no structural brain abnormalities. In contrast, three of the seven mutation-negative patients also had brain abnormalities, such as Dandy-Walker variant (*n* = 1) or aqueduct stenosis with hydrocephalus (*n* = 2). The brain MRI findings of the remaining four patients were normal.

## Discussion

In this study, we aimed to characterise the mutation spectrum of patients with macrocephaly and DD/ASD. Among the 21 patients, ten had mutations in the PI3K-AKT-mTOR signalling pathway, indicating the importance of this pathway in macrocephaly with DD/ASD (Table 2). Our overall diagnostic yield was 47.6%, and *PTEN* mutations were detected in 19% of patients (*n* = 4), similar to previous studies detecting *PTEN* mutations/deletions in patients with DD/ASD [20, 21, 48, 49]. The higher diagnostic yield in this study is because multiple genes in the PI3K-AKT-mTOR pathway in addition to *PTEN* were considered, and in selected patients, WES of reasonably high depth was performed using additional sources of DNA, including saliva or buccal mucosa samples, rather than blood samples alone. Findings from our study suggest the need to refine the recommendation of current guidelines for the genetic evaluation of patients with macrocephaly and DD/ID/ASD. The American Academy of Pediatrics guidelines do not specifically mention an evaluation of children with macrocephaly and DD/ID [50], but the ASD evaluation proposed by the Autism Consortium Clinical Genetics/DNA Diagnostics Collaboration [22], the American College of Medical Genetics and Genomics [23] and other experts [24] only suggests genetic testing for *PTEN* mutations. From a practical perspective, children with DD/ASD will be referred for genetic consultation when their developmental problem is moderate to severe or when they present with dysmorphic features. However, based on our findings, most patients with mutations present with mild to moderate developmental problems, and dysmorphism is mild and non-specific. The absence of typical features of *PTEN* hamartoma tumour syndrome (such as glans penis pigmentation, mucocutaneous lesions and lipomas) may be due to the relatively young age of the patients or to the variable presentation of these features. Thus, genetic tests should be considered for patients with DD/ASD and macrocephaly, regardless of the degree of DD/ASD and the presence/absence of dysmorphic features. A panel of genes in the PI3K-AKT-mTOR pathway, including but not limited to *PTEN*, should be tested, and a low level of mosaicism for variants should be considered when collecting samples from patients for DNA extraction and determining the methodology to use to detect mutations [25–28]. Additional sources of DNA obtained from the buccal mucosa, saliva or brain (if available) should also be used for sequencing, and next-generation sequencing with reasonably high depth and coverage of genes in the PI3K-AKT-mTOR pathway should be performed. Although our use of WES successfully identified somatic mutations in two patients, a targeted gene panel has the advantage of higher depth than WES and therefore is a better choice for testing.

The genetic diagnosis of mutations in genes involved in the PI3K-AKT-mTOR pathway is clinically important. First, monogenic mutations in the PI3K-AKT-mTOR pathway are important in the pathogenesis of a subset of patients with DD/ASD. The genetic information may facilitate genetic counselling and estimate the risk of occurrence. Second, the genetic diagnosis facilitates a determination of prognosis. For example, patients with *PPP2R5D* mutations are expected to have poor language and locomotor performance, moderate to severe ID/DD and epilepsy [29, 46]. Third, Riviere et al. recommended that brain MRI should be performed on these children, with special attention to abnormal patterns of headache, changes in gait or other neurological problems [25]. Fourth, long-term cancer surveillance should be provided for these patients, because the PI3K-AKT-mTOR pathway is an important cancer-related pathway and is frequently mutated in tumours [51]. Patients with *PTEN* mutations have an increased risk of breast cancer, thyroid cancer, melanoma and endometrial cancer [52, 53], and recently, Peterman et al. found that patients with somatic *PIK3CA* mutations had an increased risk of Wilms tumour [54]. Finally, genetic counselling and family cascade testing should be provided to patients with germline mutations, because mutations may have been inherited from parents with macrocephaly yet without a remarkable history of DD/ID. One of the *PTEN* mutations in patient 3 was maternally inherited, but his mother had a clinically unremarkable presentation, except for macrocephaly. She was counselled, and after a year of cancer surveillance, she was diagnosed with early-stage thyroid cancer. This finding illustrates the importance of family cascade testing and cancer surveillance. Nevertheless, because of the complexity of genetic tests (such as the choice of tissue and depth of sequencing) and diverse clinical presentations in patients, we emphasise that genetic testing should only be offered by clinical geneticists who provide comprehensive pre- and post-test counselling to ensure the quality of the test, data interpretation and standard of care.

Here, we reported a second patient with bi-allelic germline *PTEN* mutations. Although one of the mutations was classified as variant of unknown significance, his clinical presentation was more severe than typical patients with heterozygous *PTEN* mutations and siblings with homozygous mutations, as reported by Schwerd et al. [55]. According to these authors, the homozygous p.Leu182Ser mutation is functionally hypomorphic, and thus, the patients have a recessive form of macrocephaly syndrome with a milder clinical course and lower risk of malignancy. Our patient (patient 3) serves as a contrasting example, showing that patients with bi-allelic *PTEN* mutations can present with a more severe clinical course involving multiple systems and display early lethality.

Historically, different nomenclatures have been used in this group of patients, including but not limited to macrocephaly-capillary malformation [56], MCAP [25], MPPH [25, 30], hemimegalencephaly [26], focal cortical dysplasia [28], megalencephaly [28, 57], and *PIK3CA*-related overgrowth spectrum [58]. The overlapping phenotypic presentation makes a differential diagnosis difficult, and the use of different nomenclatures is confusing to clinicians and patients. For example, MCAP and MPPH are usually associated with *PIK3CA* and *PIK3R2* mutations, respectively. However, patients 1 and 9 in our study, who had somatic *PIK3CA* mutations, did not present with somatic features observed in MCAP other than syndactyly [47], whereas patients 3, 4 and 5 who did not have *PIK3R2* mutations, presented with megalencephaly, polymicrogyria or ventriculomegaly, consistent with MPPH. Thus, a differential diagnosis is difficult, and the clinical presentation is a spectrum. A consensus on the nomenclature for this group of patients should be reached among international clinicians and scientists to facilitate communication, management, determination of prognoses, and further research and clinical trials [58]. Although the umbrella term “*PIK3CA*-related overgrowth spectrum” has been proposed to encompass patients with *PIK3CA* mutations [58], it is not sufficiently comprehensive to describe patients with macrocephaly who are complicated with DD/ID/ASD, because mutations in genes other than *PIK3CA* have also been identified in this group of patients. Since these patients share overlapping phenotypes and mutations in the same pathway, we propose the umbrella term “mTOR pathway-related macrocephaly spectrum” to encompass patients with macrocephaly and DD/ID/ASD associated with germline or somatic mutations in the PI3K-AKT-mTOR signalling pathway.

The limitation of the present study is that we only included a small number of patients, and long-term follow up was not available for all patients. In addition, the sequencing strategy was not uniform throughout the study because we made changes to improve the sequencing depth and to include DNA obtained from saliva and buccal mucosa samples, in addition to blood samples. We believe that brain MRI findings may be an indicator for genetic testing because all patients with mutations in the PI3K-AKT-mTOR pathway had features of megalencephaly and/or brain overgrowth, rather than macrocephaly alone. Nevertheless, our findings should be confirmed in larger studies, given our small sample size. Because the association between the PI3K-AKT-mTOR pathway and macrocephaly and DD/ID/ASD is relatively new, we have limited knowledge of this disease spectrum. We hope that the identification of more patients will enable the better characterisation of the clinical presentation of this group of diseases, and hence, clinicians will be able to provide better clinical management for these patients.

## Conclusions

In summary, nearly 50% of children with macrocephaly and developmental delay/ASD had mutations in the PI3K-AKT-mTOR pathway, suggesting the importance of this pathway in this patient group. The presence of somatic mosaicism increases the difficulty in providing a molecular diagnosis, and therefore, DNA samples from different tissues should be sequenced. Finally, we propose the use of the umbrella term “mTOR pathway-related macrocephaly spectrum” to emphasise the overlapping clinical phenotypes and genotypes associated with this spectrum of patients.

## Additional files

Additional file 1: Summary of sequencing data for all patients with macrocephaly and DD/ASD. The data consist of the sequencing information for all patients recruited in this study, including the kit used to prepare the library, sequencers, and the depth of coverage. (XLSX 18 kb)

Additional file 2: Bi-allelic *PTEN* mutations in patient 3. The file consists of WES reads and clonal sequencing data to show that patient 3 has two mutations in different *PTEN* alleles. (DOCX 936 kb)

## Abbreviations

ASD: Autism spectrum disorder
COSMIC: Catalogue of Somatic Mutations in Cancer
DD: Developmental delay
DKCAC: Duchess of Kent Children’s Hospital Child Assessment Center
DQ: Developmental quotient
ExAC: Exome Aggregation Consortium
GDD: Global developmental delay
ID: Intellectual disability
MCAP: Megalencephaly-capillary malformation syndrome
MPPH: Megalencephaly-polymicrogyria-polydactyly-hydrocephalus syndrome
MRI: Magnetic resonance imaging
SD: Standard deviation
WES: Whole-exome sequencing
