# Supplementary material for: Identification of mutations in the PI3K-AKT-mTOR signalling pathway in patients with macrocephaly and developmental delay and/or autism
Source: Mol Autism. 2017 Dec 20;8:66. doi: 10.1186/s13229-017-0182-4 (PMC5738835; doi:10.1186/s13229-017-0182-4)
Supplement: Supplementary file 2 — Bi-allelic PTEN mutations in patient 3. The file consists of WES reads and clonal sequencing data to show that patient 3 has two mutations in different PTEN alleles. (DOCX 936 kb) [file 13229_2017_182_MOESM2_ESM.docx]

**Identification of Mutations in the PI3K-AKT-mTOR Signalling Pathway in Patients with Macrocephaly and Developmental Delay and/or Autism**

Kit San Yeung† ^1^, Winnie Wan Yee Tso† ^1,2^, Janice Jing Kun Ip^3^, Christopher Chun Yu Mak^1^, Gordon Ka Chun Leung^1^, Mandy Ho Yin Tang^1^, Dingge Ying^1^, Steven Lim Cho Pei^1^, So Lun Lee^,1,2^, Wanling Yang^1^, Brian Hon-Yin Chung^1,2^

1) Department of Paediatrics and Adolescent Medicine, The University of Hong Kong, Hong Kong

2) Department of Paediatrics and Adolescent Medicine, The Duchess of Kent Children’s Hospital, Hong Kong

3) Department of Radiology, Queen Mary Hospital, Hong Kong

† Equal contributors

*
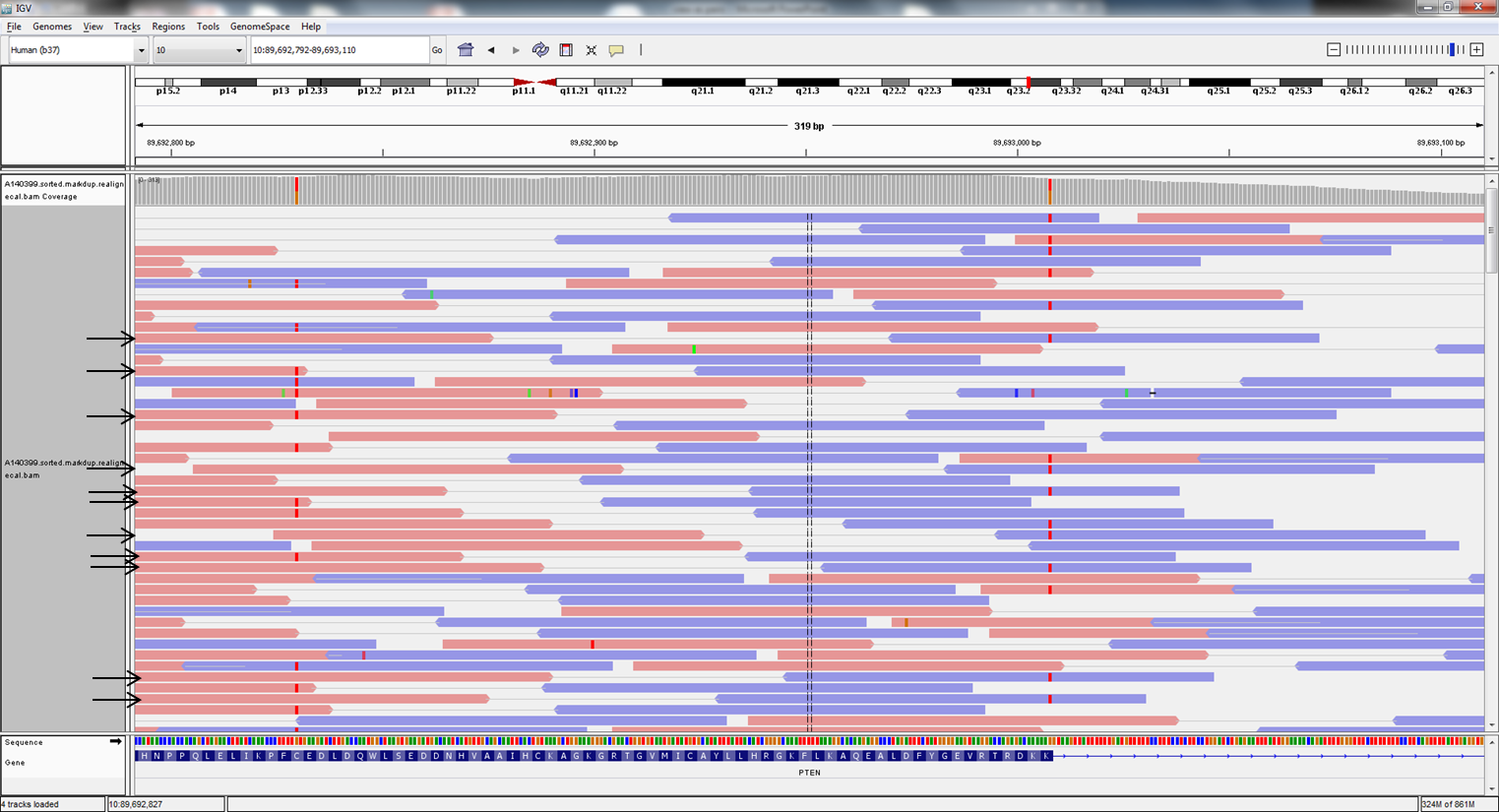
*

**Figure S1a: IGV of *PTEN* exon 5 in patient 3**Sequencing reads were viewed as pairs. Informative reads showing that the two mutations occurred in different alleles were highlighted with arrows.


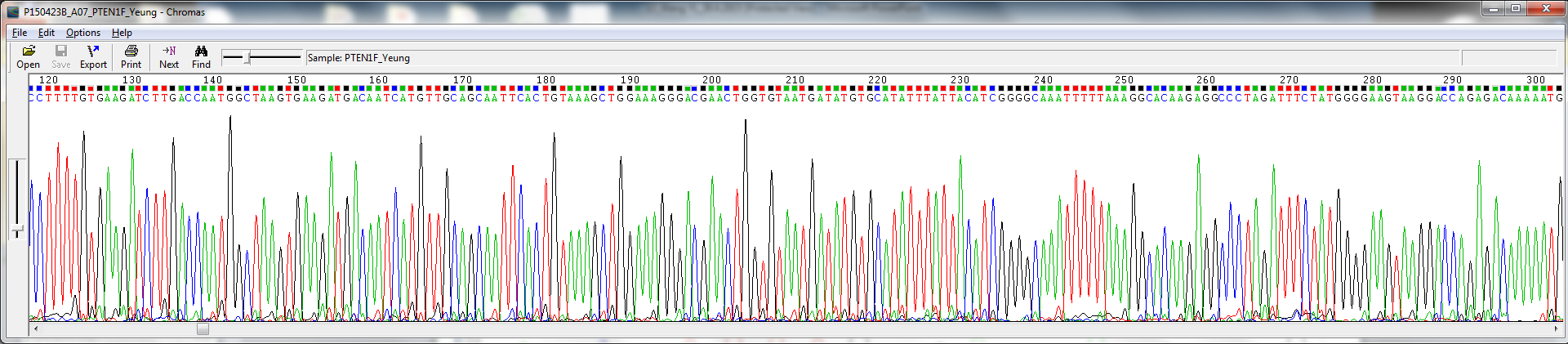

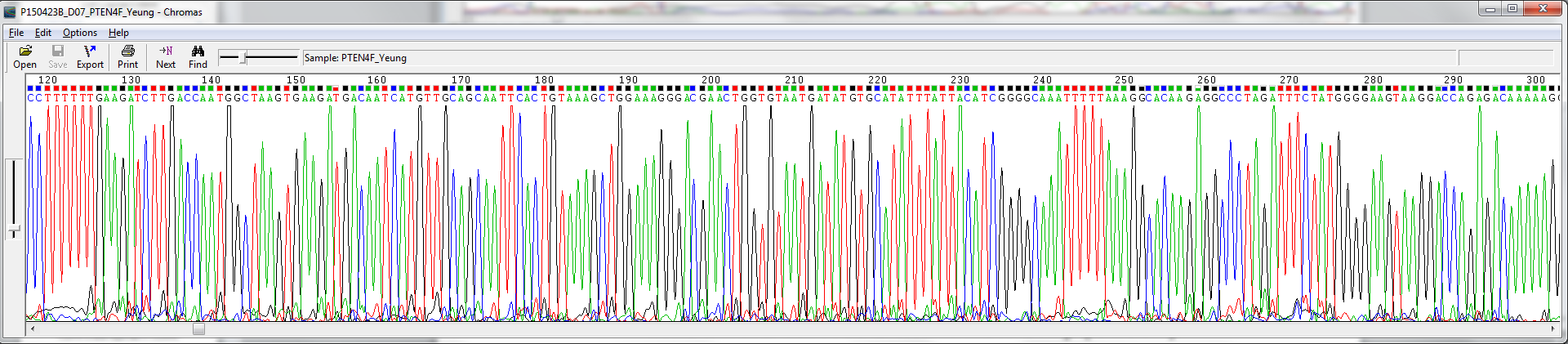


*PTEN* c.G314
(p. Cys105)

*PTEN* c.G492
(p.Lys164)

**Figure S1b: Sequencing result of cloning of *PTEN* flanking the two mutations in patient 3**Upper panel: there are only c.492G>T but not c.314G>T, indicating that this allele only has mutation of p.(Lys164Asn)
Lower panel: there are only c.314G>T but not c.492G>T, indicating that this allele only has mutation of p.(Cys105Phe)
